# Lanthanide-Loaded Nanoparticles as Potential Fluorescent and Mass Probes for High-Content Protein Analysis

**DOI:** 10.3390/bioengineering6010023

**Published:** 2019-03-15

**Authors:** Worapol Ngamcherdtrakul, Thanapon Sangvanich, Shaun Goodyear, Moataz Reda, Shenda Gu, David J. Castro, Primana Punnakitikashem, Wassana Yantasee

**Affiliations:** 1 Department of Biomedical Engineering, Oregon Health & Science University, Portland, OR 97239, USA; ngamcher@ohsu.edu (W.N.); tsangvanich@gmail.com (T.S.); goodyear@ohsu.edu (S.G.); redmo@ohsu.edu (M.R.); shendagu@gmail.com (S.G.); castro@ohsu.edu (D.J.C.); 2 PDX Pharmaceuticals, LLC, Portland, OR 97239, USA; 3 Department of Biochemistry, Mahidol University, Bangkok 10700, Thailand; primanap@gmail.com

**Keywords:** lanthanide, nanoparticle, imaging probe, mass cytometry, protein analysis

## Abstract

Multiparametric and high-content protein analysis of single cells or tissues cannot be accomplished with the currently available flow cytometry or imaging techniques utilizing fluorophore-labelled antibodies, because the number of spectrally resolvable fluorochromes is limited. In contrast, mass cytometry can resolve more signals by exploiting lanthanide-tagged antibodies; however, only about 100 metal reporters can be attached to an antibody molecule. This makes the sensitivity of lanthanide-tagged antibodies substantially lower than fluorescent reporters. A new probe that can carry more lanthanide molecules per antibody is a desirable way to enhance the sensitivity needed for the detection of protein with low cellular abundance. Herein, we report on the development of new probes utilizing mesoporous silica nanoparticles (MSNPs) with hydroxyl, amine, or phosphonate functional groups. The phosphonated MSNPs proved to be best at loading lanthanides for up to 1.4 × 10^6^ molecules per particle, and could be loaded with various lanthanide elements (Ce, Pr, Nd, Sm, Eu, Gd, Tb, Dy, Ho, Er, Yb, and Lu) at relatively similar molar extents. The modified MSNPs can also load a fluorescent dye, allowing bimodal mass and fluorescence-based detection. We achieved specificity of antibody-conjugated nanoparticles (at 1.4 × 10^3^ antibodies per nanoparticle) for targeting proteins on the cell surface. The new materials can potentially be used as mass cytometry probes and provide a method for simultaneous monitoring of a large host of factors comprising the tumor microenvironment (e.g., extracellular matrix, cancer cells, and immune cells). These novel probes may also benefit personalized medicine by allowing for high-throughput analysis of multiple proteins in the same specimen.

## 1. Introduction

A major limitation to precision oncology is the lack of a high-throughput method to concurrently analyze multiple proteins from the same tissue specimen. Likewise, the ability to simultaneously evaluate the vast number of proteins that comprise the tumor microenvironment (e.g., extracellular matrix, cancer cells, immune cells, fibroblast, among others) is highly desirable to study the systems biology of cancer [1,2]. Current fluorescent modalities cannot satisfy this requirement due to the spectral overlap of fluorescent emissions [3]. Instead of fluorophores, mass cytometry and metal-based imaging (e.g., laser ablation inductively-coupled plasma mass spectrometry, LA-ICP-MS [4]) utilizes many metal isotopes as reporting probes, thereby extending the capability to analyze more biomarkers simultaneously. However, the limitation of mass-based technology is its sensitivity, which is still lower than fluorescent imaging, and thus prevents the measurement of targets with very low expression levels [4,5]. This decreased sensitivity is due to the low number of metal molecules (about 100 metal reporters) per antibody molecule [6]. Therefore, probes carrying a larger number of metal molecules are needed. 

Lanthanides are appealing reporters because of the large number of resolvable isotopes and low natural abundance in biological/cellular systems, leading to low background signal. Additionally, lanthanides have similar chemistry, which facilitates their incorporations into the same chemical structure. Our team has previously developed a mesoporous silica microparticle material (SAMMS—Self-Assembled Monolayers on Mesoporous Supports) that has a high capacity to capture lanthanides. We have reported its use in the selective removal of lanthanides from natural water and other biological matrices [7]. Separately, we have also developed antibody-conjugated mesoporous silica nanoparticles (MSNPs) that allow the targeted delivery of therapeutics to cancer cells with high specificity [8]. Utilizing various silica surface chemical modifications, we loaded lanthanides into MSNPs prior to antibody conjugation to develop a series of mass probes for metal-based cytometry. We also loaded a fluorescent dye on the nanoparticles, thereby allowing for bimodal mass and fluorescent detections. 

Lanthanide-doped nanoparticles (e.g., NaYF4:Yb,Er/Tm) have been used as photo-luminescence probes because lanthanides can convert low-energy photons to high-energy emissions [9]. Lanthanide-doped nanoparticles have also been investigated as drug delivery carriers because they can be activated by near-infrared (NIR) irradiation to release drug cargos (e.g., spatially controlled release) [10,11]. These particles typically require high lanthanide contents and are prepared via thermal decomposition and hydrothermal/solvothermal methods, which typically require further surface modifications to render the materials hydrophilic [9]. In our application, the mass (not bioluminescence property) of lanthanides are used as the reporter. Lanthanides are loaded by covalent bonding with ligands on our mesoporous silica surface. The material is already hydrophilic, and can be further modified with antibodies for protein recognition.

## 2. Materials and Methods

### 2.1. Reagents and Cell Lines

Tetraethyl orthosilicate (TEOS), cetyltrimethylammonium bromide (CTAB), 3-(trihydroxysilyl) propyl methylphosphonate, and aminopropyltriethoxy silane (APTS) were purchased from Sigma-Aldrich Co. (St Louis, MO, USA). Branched-polyethylenimine (PEI, 10 kDa) was purchased from Alfa Aesar (Ward Hill, MA, USA). Maleimide-PEG (5 kDa)-NHS was purchased from JenKem Technology USA (Plano, TX, USA). Trastuzumab (Herceptin®, Genentech) and cetuximab (Erbitux®, Eli Lilly) were obtained from the OHSU pharmacy (Portland, OR, USA). Phosphate-buffered saline (PBS) (pH 7.2) was obtained from Life Technologies (Carlsbad, CA, USA). Zeba spin desalting columns (MW 40 kDa), RNase-free water, Traut’s reagent, ethanol, HCl, NHS-rhodamine, and sodium hydroxide were purchased from Thermo Fisher Scientific (Waltham, MA, USA). All reagents were of the highest purity grade available. Cell lines (MDAMB468, BT549, MDAMB231, KPL4 and MCF7) were obtained from American Type Culture Collection and maintained following the supplier’s recommendation.

### 2.2. Nanoparticle Synthesis and Characterization

MSNPs were synthesized by a sol–gel method, following prior publications [8,12] with modifications. In brief, mesoporous silica nanoparticles (MSNPs) were formed by the addition of TEOS (1.2 mL) to the solution of 6 mM CTAB (120 mL, pH 11 adjusted by 2 M NaOH) at 80 °C under stirring. Twenty minutes after TEOS was added to the surfactant solution, 0.6 mmol of 3-(trihydroxysilyl) propyl methylphosphonate was added for the phosphonated surface. For the aminated surface, 0.6 mmol of aminopropyltriethoxy silane (APTS) was added. Without co-precipitation of organosilanes, MSNPs have a hydroxyl surface. The reaction continued for 2 h total for each construct. More uniform MSNPs were achieved by replacing CTAB with 0.15 M cetyltrimethylammonium chloride and 0.022 M triethanolamine [8]. The MSNPs were then pelleted down by centrifugation, washed with a copious amount of ethanol, and dried overnight. MSNPs were then refluxed in acidic methanol to remove surfactant in the sol–gel synthesis. For the dye-tagged construct, 10 mg of aminated MSNPs was reacted with 1 mg of rhodamine via N-hydroxysuccinimide (NHS) reaction. Both MSNPs and dye-tagged MSNPs were washed with ethanol and dried in a desiccator until ready for use. A large excess of rhodamine for nanoparticle loading was used in order to achieve maximal rhodamine loading (which should translate to maximal signal per binding event of each nanoparticle).

MSNPs were decorated with antibodies (i.e., trastuzumab or cetuximab) following our prior published work [8]. In brief, the MSNPs were conjugated with NHS ester-polyethyleneglycol (PEG)-maleimide via the amine groups on MSNPs. Ten milligrams of aminated MSNPs was mixed with 10 mg mal-PEG-NHS ester for 2 h. For MSNPs without amine groups, we pre-coated the particles with ~15 wt.% polyethylenimine (PEI) to provide amine groups. PEI-MSNP (10 mg) was then mixed with 10 mg mal-PEG-NHS ester for 2 h. The PEGylated particles were then centrifuged and washed three times with PBS (pH 7.1). 

Concurrently, the antibodies were thiolated for 2 h by mixing 0.8 mg of Traut’s reagent and 10 mg of antibody in 5 mL of PBS (pH 8.0) at room temperature. Thiolated antibody was purified using a Zeba spin column (40 kDa) and conjugated to the PEGylated nanoparticle at an antibody: MSNP mass ratio of 1:10 via the thiol-maleimide reaction under shaking overnight at 4 °C. Final antibody-conjugated nanoconstructs were centrifuged and washed with PBS (pH 7.1), and characterized for size and charge by dynamic light scattering (Zetasizer). The compositions were analyzed with the thermogravimetric (TGA) and bicinchoninic acid (BCA) assay following our prior works [8,13,14].

### 2.3. Lanthanide Loading on the Nanoparticles and Loading Characterization

The MSNPs were loaded with lanthanide elements in an aqueous solution (5 mg/mL MSNPs in 2000 mg/L of lanthanide elements in water) for 2 h. The amount of lanthanide loaded on the MSNPs was determined by the difference of lanthanide content in the supernatant before and after MSNP loading and confirmed by 10 M HNO_3_ acid leaching of lanthanide-containing MSNPs. The amount of lanthanide in solution was determined by ICP-MS analysis. For multi-element loading, 0.2 mg/mL MSNPs was reacted with a mixture of 10 µg/L (ppb) of each lanthanide element for 2 h. 

### 2.4. Imaging and Flow Cytometry

Cell suspensions (1 × 10^6^ cells/mL—e.g., BT474, MDA-MB-468, BT-549, MDA-MB-231, KPL4, MCF7) were incubated with 0.2 mg/mL of antibody-conjugated nanoparticles for 1 h. Cells were washed two times with PBS (pH 7.1) by centrifugation. The cell suspension was dropped on the coverslip for imaging using fluorescent and phase-contrast microscopy. Flow cytometry was used for quantitative nanoparticle uptake analysis of treated cells. After 1 h of contact time, cells were washed three times with repeated centrifugation (115 *g*, 5 min) with FACS buffer (1 mL, PBS (Ca/Mg^++^ free), 1 mM EDTA, 25 mM HEPES pH 7.0, 1% FBS (heat-inactivated)), then resuspended in the FACS buffer (550 μL final volume) and kept on ice until analysis. For cells stained with free antibody (for gating purposes), antibody labeling was performed on ice and under rocking conditions. Cells were stained with primary antibody (e.g., cetuximab, 2 μg per tube) for 1 h, washed (i.e., centrifuge at 115 *g*, 5 min) with PBS once, stained with secondary antibody (Anti-human Alexa488, 2 μg per tube) for 45 min, then washed two times with PBS, and resuspended in FACS buffer (550 μL final volume) before analysis. All cells were counter-stained for cellular DNA with DRAQ5 (2 μL, 5 mM) (Cell Signaling, Danvers, MA) for 15 min on ice. Then, all tubes (except antibody-labeled cells for gating purposes) were incubated with Trypan Blue (500 μL, 0.4% in PBS) to quench fluorescence outside of the cells, and subjected to flow cytometry analysis. A total of 10,000 events (cells) were analyzed for each sample. The intensity was processed with FlowJo software (FlowJo LLC, Ashland, OR).

### 2.5. Western Blot

Cells were lysed in RIPA buffer containing protease and phosphatase inhibitor cocktails, and protein was quantified using BCA assay. Samples were prepared with 4X Novex NuPAGE LDS sample buffer (Life Technologies) and supplemented with 10% beta-mercaptoethanol. Twenty micrograms of protein was loaded per lane of 4%–12% Bis-Tris NuPAGE gels (Life Technologies) and run in MOPS buffer. Following gel electrophoresis, proteins were transferred to a PVDF-FL membrane (Millipore) in tris-glycine transfer buffer. Primary antibody incubation was carried out overnight at 4 °C on a rocking platform. Incubation with IRDye-conjugated secondary antibodies was carried out at room temperature the next day. Proteins were detected using the LI-COR Odyssey imaging system (LI-COR Biosciences). Image analysis and protein quantitation (i.e., band densitometry) was performed with ImageJ (NIH, Washington, DC, USA). 

## 3. Results

### 3.1. Nanoparticle Characterization

**Figure 1:**
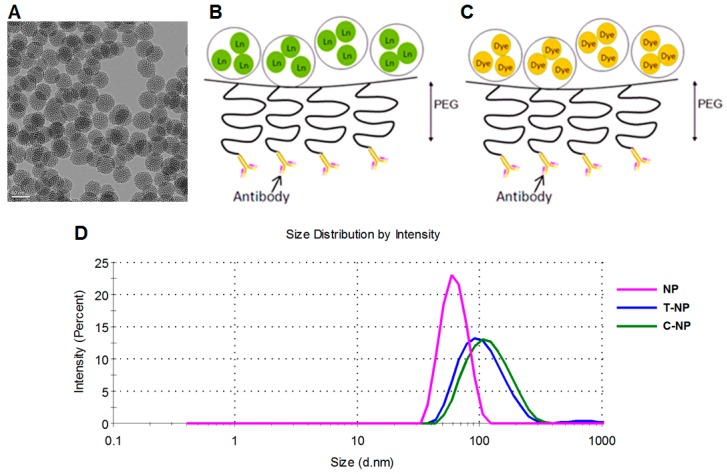
(**A**) TEM image of the mesoporous silica nanoparticle (MSNP, hydroxyl) core (scale bar = 50 nm) and (**B**,**C**) illustrations of NPs coated with polyethylene glycol (PEG) which avoids non-specific binding of nanoparticles and serves as a linker for NPs and antibody. Both lanthanide (Ln) and fluorescent dye can be loaded inside the pores of MSNPs, as illustrated in (**B**,**C**), respectively, prior to surface modification. (**D**) Hydrodynamic sizes before and after surface modification with trastuzumab (T-NP) or cetuximab (C-NP).

Mesoporous silica nanoparticles (TEM size of ~50 nm) were synthesized by sol–gel method, following our prior published protocol [8]. FTIR spectroscopy was used to confirm the absence of surfactants and residual reagents, as reported in our prior work [8]. MSNP core size by TEM was 47 ± 4 nm as shown in Figure 1A. Lanthanides and fluorescent dye could be loaded into the MSNP pores as illustrated in Figure 1B,C, respectively. The hydrodynamic size of MSNPs was 63 ± 1 nm, which increased to 106 ± 1.3 nm after polymer loading and antibody conjugation (Figure 1D). Surface modification was performed layer by layer, making it easy to confirm successful loading by weight (via thermogravimetric analysis [8]) and by zeta potential analysis. Specifically, the MSNPs had a zeta potential of -20.6 ± 1.1 mV, which increased to 42.5 ± 1.4 mV after amine loading, and reduced to 17.8 ± 0.3 mV after PEG attachment. All zeta potential values were measured in 10 mM NaCl. The polymer and antibody contents were reported in our prior publication [8] by TGA analysis to be 13.5 wt.% for PEI, 18.2 wt.% for PEG, and 3 wt.% for antibody.

### 3.2. Lanthanide Loading Capability of Our Nanoparticles

MSNPs with different functional groups were loaded with thulium (Tm) as a model lanthanide. Tm was selected because it has only one isotope (^169^Tm with 100% abundance); 5 mg/mL NPs was mixed in 2000 mg/L (ppm) Tm. In agreement with our prior work on lanthanide-binding microparticles [7], Table 1 shows that MSNPs with a phosphonated surface absorbed the highest amount of Tm compared to aminated and hydroxyl surfaces. 

**Figure 2:**
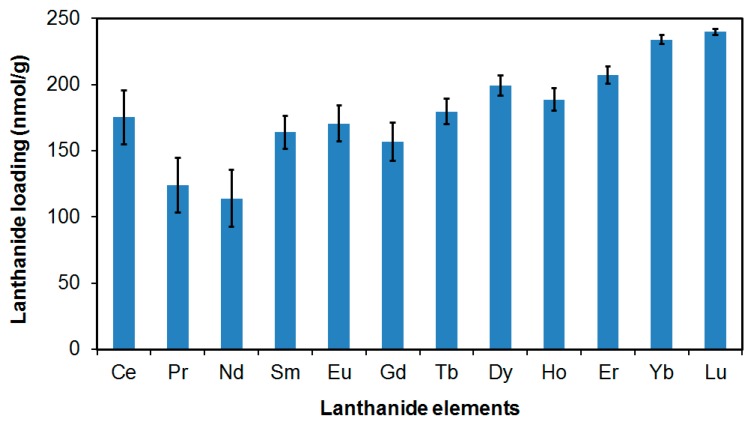
Loading of lanthanide elements on phosphonated MSNPs. A cocktail of lanthanides at 10 µg/L each was in contact with 0.2 mg MSNPs for 2 h.

The maximum Tm loading of 32.4 mg/g was equivalent to 0.192 mmol/g or 1.2 × 10^20^ molecules/g. There were 8.8 × 10^13^ nanoparticles per gram of nanoparticles (NP counts measured with Nanosight system (CA)). Thus, the maximum lanthanide loading was 1.4 × 10^6^ molecules per nanoparticle. We next loaded the phosphonated MSNP core with a cocktail of lanthanides as shown in Figure 2. We found phosphonated MSNPs to be versatile and capable of carrying any lanthanide elements at relatively similar molar extents (179 ± 38 nmol/g). Notably, the loading extents were low in Figure 2 compared to those in Table 1 because we used only 10 ppb of the starting lanthanide concentrations.

In terms of lanthanide binding stability, we have extensively studied lanthanide binding on three phosphonate functional groups on mesoporous silica (in micron size), mainly for water treatment application [7]. Lanthanide binding on phosphonate-modified mesoporous silica is very strong at conditions similar to physiological conditions (e.g., pH 7–8 with high chloride and bicarbonate ion contents). At these conditions, the materials covalently captured multiple lanthanides by 10^5^–10^7^-fold (by mass) of lanthanides left in the solution. Only when the pH was reduced to below 2.5 (i.e., outside of this operating range) did leachate of some lanthanides occur. Nevertheless, the leachate of these probes will be assessed in our follow-up studies.

### 3.3. Antibody-Conjugated Nanoparticles Bind to Cells Expressing Target Proteins with High Specificity

**Figure 3:**
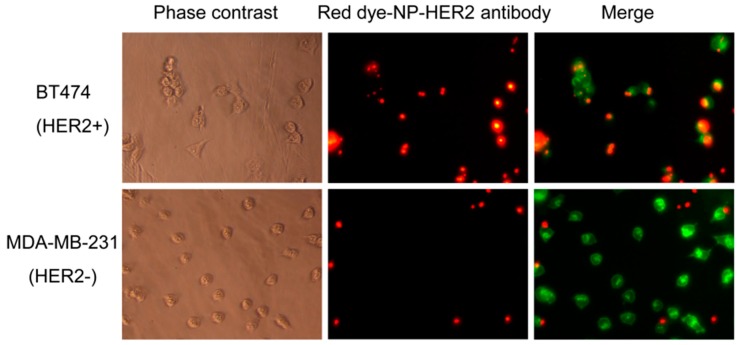
Microscopic images of cell labeling of trastuzumab-conjugated rhodamine-MSNPs (red) for BT474 (HER2+ cells) and MDA-MB-231 (HER2- cells). Cells were counter-stained with phalloidin (green). **Left** column: Phase contrast images; **Middle** and **Right** columns: Fluorescence images (20× magnification).

As a proof-of-concept, we conjugated the MSNPs with rhodamine dye-NHS ester and trastuzumab (a clinical-grade anti-HER2 antibody). Antibody loading was 3 wt.% of the nanoparticles, as determined by BCA assay. Since the molecular weight of trastuzumab is 145,531 g/mol and there were 8.8 × 10^13^ nanoparticles per gram of nanoparticles, the antibody loading was 1.4 × 10^3^ molecules per nanoparticle. Figure 3 shows that the nanoparticles bound preferentially to HER2+ breast cancer cells (BT474) compared to HER2- breast cancer cells (MDA-MB-231). This agrees with our previous report that the trastuzumab-conjugated MSNPs were preferentially taken up by HER2+ cells over HER2- cells [8]. 

**Figure 4:**
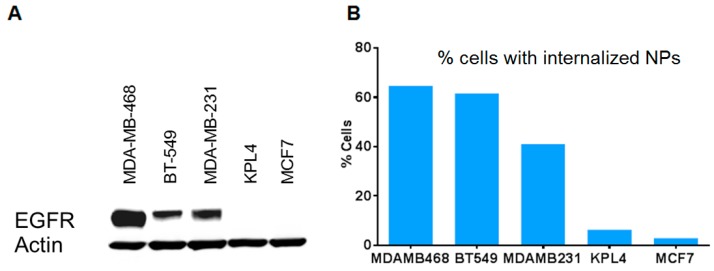
Specific uptake of cetuximab (anti-EGFR)-conjugated nanoparticles to EGFR+ vs. EGFR- breast cancer cells. (**A**) EGFR expression of cells by Western blot analysis. (**B**) Flow cytometry analysis of cells taking up dye-tagged nanoparticles after 1 hr of contact time.

We also conjugated the nanoparticles with cetuximab (a clinical-grade anti-EGFR antibody). The nanoparticles were preferentially taken up into EGFR+ cancer cells (MDA-MB-468, BT-549, MDA-MB-231) over cells that were EGFR- (KPL4, MCF7), as analyzed by flow cytometry (Figure 4). This indicates the versatility of the probes for targeting many proteins upon switching antibodies.

## 4. Discussion

Our data show the great potential of antibody-conjugated MSNPs loaded with lanthanides and dyes as reporter probes for mass cytometry and fluorescence-based imaging. We showed that phosphonated MSNPs could carry the highest amount of lanthanide (thulium, 32 mg/L), compared to unmodified MSNPs and aminated MSNPs. The nanoparticles were also versatile and could load various lanthanide elements at relatively similar molar extents. Concurrently, the antibody-conjugated dye-loaded nanoparticles could specifically bind to target proteins on the target cells. Together, these preliminary findings suggest great feasibility for this MSNP technology to be used as a highly sensitive reporter for mass cytometry.

The current standard for mass probe is a chelating polymer-conjugated antibody that only carries a maximum of around 100 metal ions [6]. Another promising alternative is a nanoparticle made entirely of lanthanide [15], which can substantially amplify the lanthanide signal on the antibody. The former method is more flexible because selected lanthanides can be loaded on the ready-made carriers without having to synthesize different types of lanthanide nanoparticles, as in the latter case. Additionally, different lanthanide nanoparticles may have different surface chemistries and sizes, leading to less detection robustness across different lanthanide probes. Herein, we propose an alternative approach that offers the versatility of the reporter particles (i.e., capable of carrying any type of lanthanide), while also having substantial lanthanide loading capacity (~10^6^ molecules/MSNP).

Our follow-on work will evaluate the lead nanoparticle probes in cells with mass cytometry (CyTOF) [16] and in tissues with mass-spectrometry immunohistochemistry [17] or LA-ICP-MS [4]. Using a library of lanthanides and their isotopes, mass cytometry has the ability to resolve many reporter tags at the same time, thus allowing for a high-throughput, and high-content approach for diagnostic assays. This nanoparticle technology holds great promise to improve the needed sensitivity of mass cytometry for protein analysis of biospecimens.

## Figures and Tables

**Table 1:** Different surface functionalities vs. thulium loading.

MSNP Surface Functionality	Tm Loading (mg Tm/g Material)
Hydroxyl	14.6 ± 0.6
Aminated	25.3 ± 1.0
Phosphonated	32.4 ± 1.7
